# Timing of fluorodeoxyglucose positron emission tomography maximum standardized uptake value for diagnosis of local recurrence of non‐small cell lung cancer after stereotactic body radiation therapy

**DOI:** 10.1002/cam4.3302

**Published:** 2020-08-26

**Authors:** Daren Tan, Suki Gill, Nelson Loh

**Affiliations:** ^1^ Department of Radiation Oncology Sir Charles Gairdner Hospital Perth WA Australia; ^2^ Department of Nuclear Medicine Sir Charles Gairdner Hospital Perth WA Australia

**Keywords:** CT PET, FDG‐PET, NSCLC, radiosurgery, standardized uptake value

## Abstract

**Introduction:**

After treatment with stereotactic body radiation therapy (SBRT), local recurrence of non‐small cell cancer (NSCLC) can be difficult to differentiate from radiation‐induced changes. Maximum standardized uptake value (SUVmax), measured with 18‐F‐Fluorodeoxyglucose positron emission tomography (FDG‐PET), can have false positives due to acute radiation inflammation. The primary study objective was to determine the utility of SUVmax > 5 to identify local recurrence later than 9 months after SBRT.

**Method:**

A retrospective review was performed of FDG‐PET scans for suspicious CT findings after SBRT treatment of stage 1 NSCLC. SUVmax was measured including surrounding opacification. Outcome measures were local recurrence, progression free survival, and overall survival. Receiver operator curve analysis, sensitivity, specificity, and Kaplan‐Meier analysis were performed.

**Results:**

Of 118 patients treated, 42 patients had eligible FDG‐PET scans. They received SBRT (48‐60Gy in 3‐8 fractions) for 49 NSCLC and had 101 follow‐up PET scans. The median time to first PET scan was 9.3 months, and the median follow‐up period was 22.4 months. Local recurrence was diagnosed in 12 patients, at a median of 16 months. Due to selection bias, the included patients had poorer outcomes than the entire cohort, with progression free survival (PFS) at 1, 2, and 3 years of 82.7%, 57.8%, and 45.8%; and overall survival of 97.9%, 79.9%, and 59.1%, respectively. Thirty FDG‐PET scans were performed within 9 months, of which 17% were false positives. A total of 71 FDG‐PET scans were performed beyond 9 months, and the median SUVmax was significantly higher for patients with local recurrence (7.48 vs. 2.14, *P* < .0001). SUVmax > 5 has a sensitivity of 91% (95% CI 62%‐99.8%) and 100% (89.1%‐100%).

**Conclusion:**

For local recurrence of NSCLC, SUVmax > 5 on FDG‐PET scan has good sensitivity and specificity after 6 months, but is highest beyond 9 months after SBRT.

## Graphical abstract

A retrospective review of 49 NSCLC after stereotactic radiotherapy, with 2 years followup and PET scans. For local recurrence of NSCLC, PET scan is best beyond 6‐9 months after SBRT. SUVmax > 5 has a sensitivity of 91% (95% CI 62%‐99.8%) and 100% (89.1%‐100%).

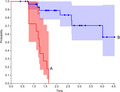

## 1 INTRODUCTION

Lung cancer is the most frequent cancer worldwide, and a leading cause of cancer death.^1^ Stage I non‐small cell lung carcinoma (NSCLC) is defined by the American Joint Commission on Cancer (AJCC) as a T1 or T2 tumor in the parenchyma of the lung, no more proximal than 2cm from the carina, and not invading chest wall or parietal pleura.^2^ Current recommendations from ASTRO and ESTRO are that patients with inoperable T1‐2a N0 NSCLC with tumors less than 5 cm in diameter are appropriate for SBRT following discussion at a multidisciplinary team meeting, and this treatment may be considered curative.^3^, ^4^ SBRT is a viable treatment option for early‐stage NSCLC, with meta‐analysis of nonrandomized studies finding local control and disease free survival approaching that of surgery.^5^, ^6^

Follow‐up is important to ensure that appropriate salvage treatments can be considered, and active surveillance is recommended.^3^, ^4^ Disease recurrences after SBRT occurs in two distinct patterns. The predominant pattern is out‐of‐field, isolated distant recurrence (46%) presenting early, despite initial PET staging.^7^, ^8^ The other pattern is isolated locoregional recurrence (34%).^7^, ^8^

Diagnosis of isolated local recurrence is important because salvage surgery, cryoablation, radiofrequency ablation, or repeat SBRT is often feasible and can significantly improve prognosis.^9^ CT imaging can be difficult to interpret as radiation‐induced lung opacities can occur in up to 91% of patients.^10^ It may not precisely correspond to the planning target volumes (PTV) and may dynamically vary both in shape and location during the follow‐up period.^10^, ^11^ Mass like fibrosis is often observed at 1‐2 years and may be difficult to distinguish from tumor recurrence, even at a chronic phase after SBRT.^9^, ^10^ This makes it difficult to assess for recurrence with the traditional Response Evaluation Criteria In Solid Tumours (RECIST).^12^

The current consensus is that FDG‐PET/CT scans are recommended in cases where there is suspicion of local, regional, or metastatic recurrence, however, there is limited evidence to guide routine use.^13^ Maximum standardized uptake value (SUVmax) is routinely measured on PET scans and provides a semi‐quantitative approximation of tumor glucose metabolism.^14^ Factors affecting SUVmax include obesity, blood glucose level, respiratory movements, camera type, and reconstruction algorithms, and it is less accurate for small lesions less than 5 mL.^15^ SUVmax corrected for lean body weight (SULmax), may confusingly also be reported as SUVmax. A variety of other measurements are based on SUV, including SUV mean, metabolic tumor volume, total lesion glycolysis, and Positron Emission Tomography Response Criteria in Solid Tumours 1.0 (PERCIST).^16^ In both preoperative and pre‐SBRT studies, pretreatment SUVmax has prognostic significance for overall survival, local control, and distant metastasis.^17^

Several studies have looked at SUVmax for local recurrence after SBRT, but it remains controversial for local recurrence due to residual activity at the treatment site caused by radiation‐induced pneumonitis, inflammation, and fibrosis.^10^, ^14^, ^18^, ^19^, ^20^, ^21^, ^22^, ^23^, ^24^, ^25^ Data available suggest that after 3‐9 months, acute radiation changes may be resolving and allowing the metabolic activity of recurrent tumors to be more reliably detected. A SUVmax threshold of 5 has been identified as useful for identifying lesions at high risk of subsequent local failure.^10^, ^11^ We assessed the utility of SUVmax > 5 on FDG PET for local recurrence after SBRT, especially beyond 9 months after treatment.

## 2 METHOD

A single‐centre retrospective review was performed of patients treated with SBRT for stage 1‐2 NSCLC between April 2014 and 2018. Inclusion criteria were NSCLC cT1/2aN0M0 (AJCC 7th edition), treated with SBRT with curative intent. Histological confirmation of NSCLC was preferred but not required. All patients had a staging FDG‐PET and were discussed at lung MDT and deemed high risk or inoperable. Patients with previous surgery, chemotherapy, or radiotherapy were eligible. Patients who had less than 6 months of follow‐up were excluded. Patients who did not have a FDG‐PET scan at our centre were excluded due to heterogeneity in PET protocol and equipment. Progression and survival data were collected for all patients. This retrospective review only included patients who had consented to their data being used for research.

SBRT was delivered using the CyberKnife® Robotic Radiosurgery System. Most patients were treated with fiducial tracking via the Synchrony® System, with a 5mm PTV margin. In a minority of patients, Spine tracking and Xsight® Lung Tracking were utilized (with margins < 10 mm) after discussion at peer review. Patients were followed up 3 monthly with CT scans for the first 2 years, and 4‐6 monthly thereafter. PET was performed at the discretion of the treating oncologist, typically after a suspicious CT scan result.

FDG‐PET imaging was performed as per departmental protocol, from midthigh to vertex. After fasting for 6 hours, 5MBq/kg of FDG was intravenously injected if the patient's blood sugar was less than 11.0mmol/L. After 60 minutes, image acquisition was performed using either a Siemens Biograph 16 or Biograph mCT64 scanner. SUVmax was not routinely reported, so was calculated for a region of interest encompassing the primary tumor and surrounding CT opacification. This was done on a Siemens Syngo.via workstation, under the supervision of a nuclear physician. SULmax was also calculated on the same workstation for the same region of interest, which corrects the SUVmax using a predicted lean body mass (default estimation based on weight, height, and gender).

This study defines local control (LC) as the absence of recurrence of tumor within 2cm of the planning target volume. Local recurrence was either histologically proven or by progressive opacity (>20%) on at least 3 serial CT studies. Local recurrence, intrathoracic lymph node recurrence (regional recurrence), and distant metastasis were calculated from the date of completion of SBRT. For patients with multiple tumors, time to recurrence was calculated from the date of the most recent treatment.

PET scans were grouped into 3‐month blocks for statistical analysis, however, due to insufficient power, analysis was repeated with scans categorized into before or after 9 months. Patients with multiple PET scans were censored at local recurrence and only included once in each time period, taking their highest SUVmax. The 9‐month cut‐off was determined post‐hoc to produce the best diagnostic accuracy. Analysis was repeated for a 6‐month cut‐off. The distributions of SUVmax with and without recurrence were compared using the Mann‐Whitney *U* and Student's T test. Kaplan‐Meier curves, Chi squared, and log‐rank tests were used to estimate the local control, progression free, and overall survival. The predictive performance of SUVmax and SULmax was assessed by receiver‐operating characteristic curves. Optimal thresholds were determined by minimum balanced error rates. The 95% intervals for sensitivity and specificity were calculated. For all tests, a *P*‐value < .05 was considered significant. Analyses were performed using Medcalc 19.3 (Medcalc software Ltd.).

## 3 RESULTS

**FIGURE 1:**
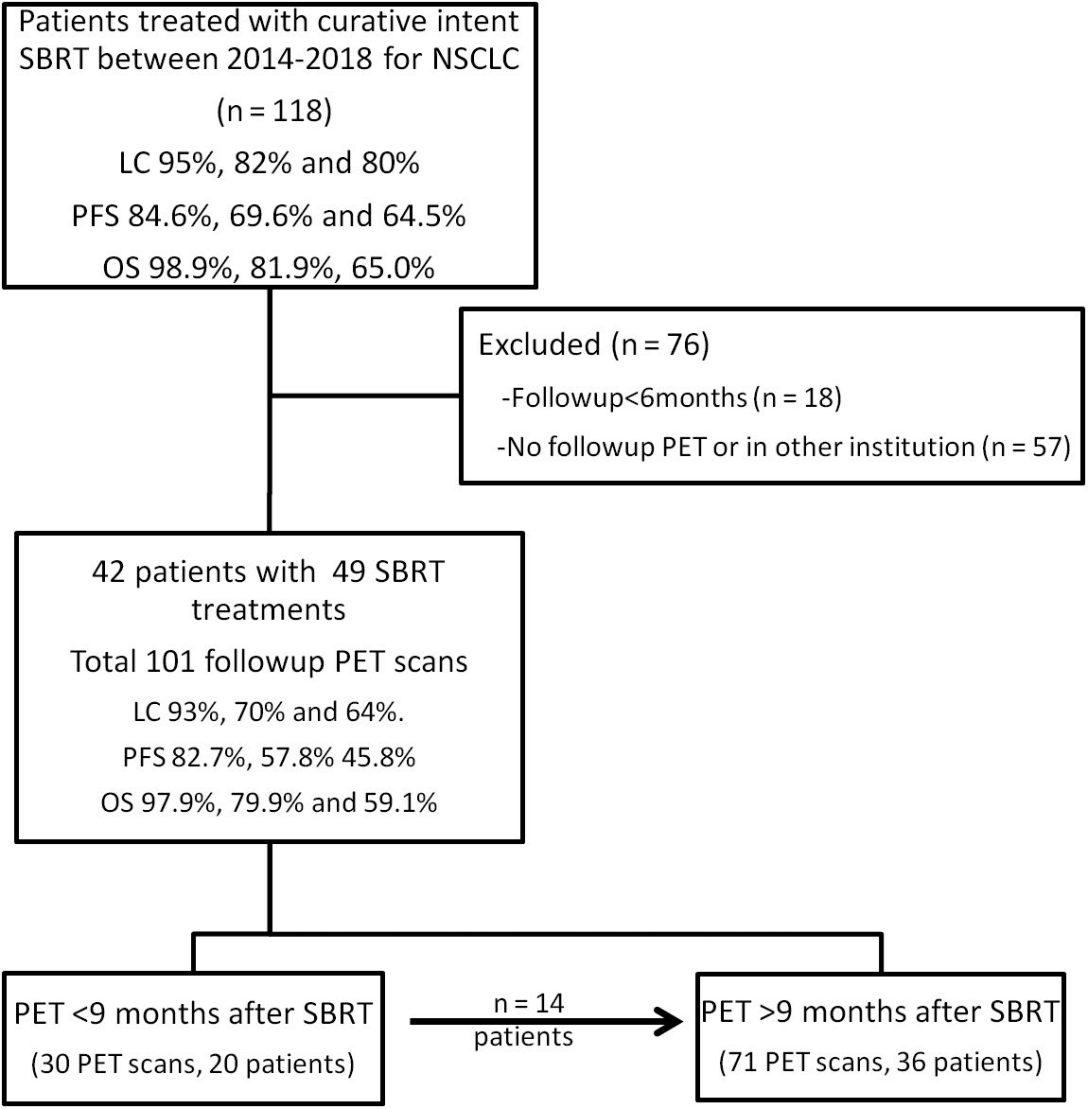
Study flow chart. Local control (LC), Progression free survival (PFS), and Overall survival (OS) survival at 1, 2, and 3 y

A total of 118 patients received 133 treatments of curative lung SBRT for NSCLC between 2014 and 2018. Nineteen patients were excluded for follow‐up less than 6 months, and 57 patients were excluded as they did not have any follow‐up FDG‐PET at our centre (See Figure 1). The remaining 42 patients received SBRT treatment for 49 lesions and were included in this study. The demographic, tumor, and treatment characteristics are detailed in Table 1. Four patients were considered to have synchronous primary tumors and were treated simultaneously, while three patients had a second primary tumor which was treated at a later stage.

**TABLE 1:** Patient characteristics

Number of participants		42
Number of NSCLC lesions treated		49
Age	Median (range)	72.8 (56‐90)
Gender	Male	30 (71%)
Stage	1	36 (73%)
	2	13 (27%)
Pathology	Adenocarcinoma	30 (61.2%)
	Squamous cell	9 (18.4%)
	Other	2 (4%)
	No pathology	8 (16.3%)
Dose/fractionation	54‐60Gy/8#	26 (62%)
	44‐48Gy/8#	3 (6%)
	45‐55Gy/5#	3 (6%)
	48/4#	4 (12%)
	54/3#	13 (27%)
Tracking	Spine tracking	7
	Fiducial tracking	22
	Tumor tracking	18
Mean PTV	T1	18.2 cm^3^
	T2	56.5 cm^3^
Follow‐up	Median	23.8
	Range	7‐55
PET	Number	101
	0‐6 mo	20
	6‐12 mo	27
	>1 y	54
Median SUVmax	Pretreatment	4.9
	Posttreatment	2.8
Median SULmax	Pretreatment	3.8
	Posttreatment	2.2
Site of first recurrences	Local	12 (28%)
	Hilar	2 (5%)
	Metastatic	5 (12%)

### 3.1 Patterns of failure

At a median follow‐up of 23.8 months (range 7.1‐54.5 months), local recurrence was found in 12 patients (10% of 118 patients) including 6 which were histologically proven. This includes 2 who were concurrently diagnosed with recurrence in the mediastinum. Except for one recurrence (which was outside but within 2 cm of the PTV), the remaining 11 local failures occurred in patients receiving 5‐8 fractions. Any recurrence occurred in 22 patients, with the site of first recurrence being: local in 12 patients (54% of recurrences), mediastinal in 5 patients (22%), at a median of 20 months (range 5‐45), and metastatic in 5 (22%) patients at a median of 9 months (range 3‐25).

The local control rates at 1, 2, and 3 years were 93%, 70%, and 64% (The corresponding rates for all 118 patients were 95%, 82%, and 80%). For local recurrences in the study population, 3 (25%) were diagnosed between 9 and 12 months, 8 (67%) from 12 to 24 months, and 1 (8%) after 24 months. Further treatment was provided for 9 patients with local recurrence: 2 patients underwent surgical resection, 4 patients had further radiation therapy, and 3 received systemic therapies.

For all 118 patients, the progression free survival (PFS) at 1, 2, and 3 years were 84.6%, 69.6%, and 64.5%, respectively, and the corresponding OS was 98.9%, 81.9%, and 65.0%. For the study population, the PFS at 1, 2, and 3 years was 82.7%, 57.8%, and 45.8%, and OS was 97.9%, 79.9%, and 59.1%, respectively. Median survival was not estimable as the Kaplan‐Meier curve never reached 50% for overall survival.

### 3.2 Timing of PET

A total of 101 posttreatment FDG‐PET scans were performed. The median time to first follow‐up PET scan was 9.3 months, and 15 months for all subsequent PET scans.

Within 9 months of SBRT, 30 PET scans were performed with a median SUV max 2.8 (range 0.9‐21). The sensitivity was 25% (0.6%‐80.6%) and specificity 89% (67%‐98%). Three patients had SUVmax > 5 (total 5 PET scans). Two of these patients were false positives, with no recurrence at last follow‐up (15 and 20 months). They had gradually decreasing SUV on serial PET, but were still greater than 5 at 7‐8 months. The third patient had a biopsy‐proven recurrence at 17months. Three patients with a low SUVmax eventually developed high SUV and biopsy‐proven local recurrence at 18‐22 months.

**FIGURE 2:**
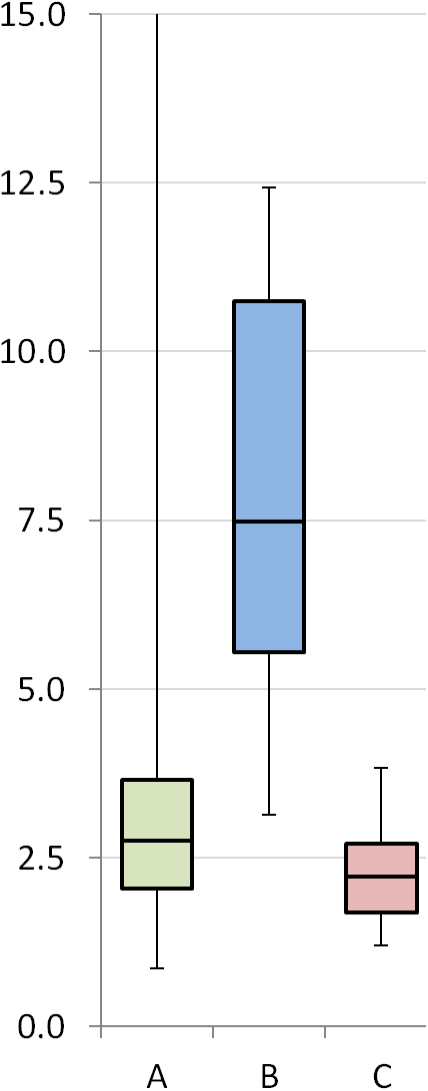
Box plot of SUVmax. A: SUVmax of PET scans performed before 9 mo. n = 30; B: SUVmax of scans > 9 mo in patients with local recurrence. n = 12; C: SUVmax of scans > 9 mo in patients without local recurrence. n = 44

A total of 71 PET scans were performed from 9 months to 44 months after treatment. There was a statistically significant difference between median SUVmax for patients with local recurrence 7.5 (range 3.4‐12.4) compared to 2.1 (range1.7‐3.8) in those without (*P* < .001) (Figure 2). The threshold SUV for local recurrence for the 71 PET scans was found to be SUVmax > 5 by the use of receiver operating characteristic curves. For this cohort of 12 local recurrences in 36 patients, SUVmax > 5 had a sensitivity of 91% (95% CI 62%‐99.8%) and specificity 100% (89.1%‐100%). There was one false negative that had a SUVmax of 3.37 at 32 months, with an enlarging infield recurrence on serial CT scans. She did not have a biopsy, and died at 42 months.

Secondary analysis was conducted for PET performed more than 6 months after treatment. The receiver operator curve produced the same SUV > 5 threshold, with a sensitivity of 91.67% (61.5%‐99.8%) and specificity of 95.1% (83.5%‐99.4%)

**FIGURE 3:**
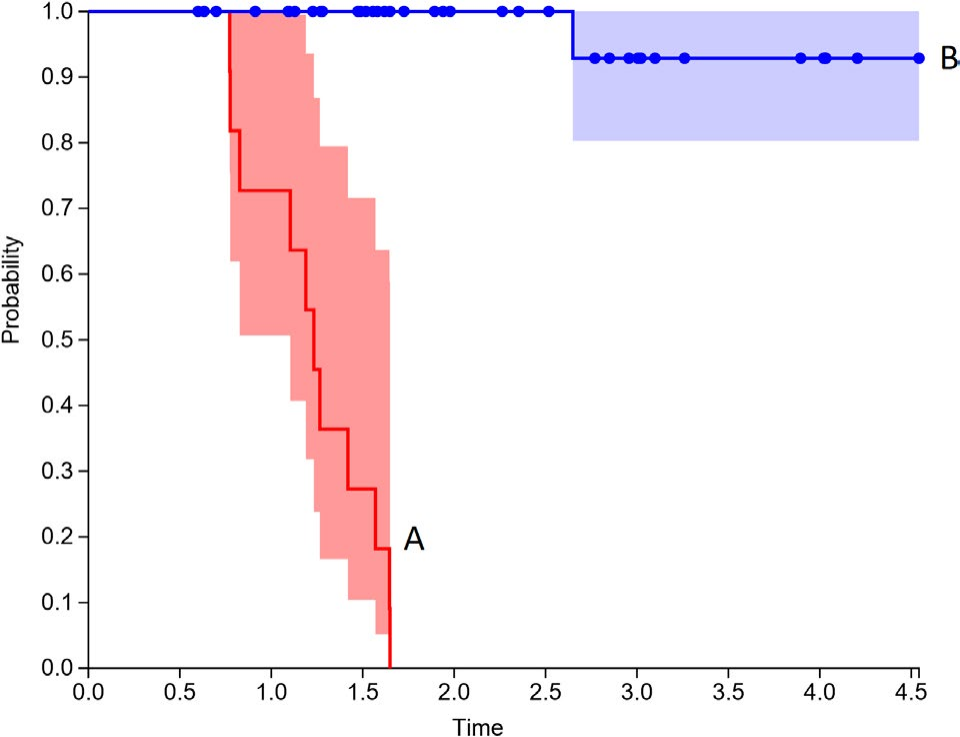
Kaplan‐Meier plot for Local control: Group A SUVmax > 5 and Group B SUVmax < 5

**FIGURE 4:**
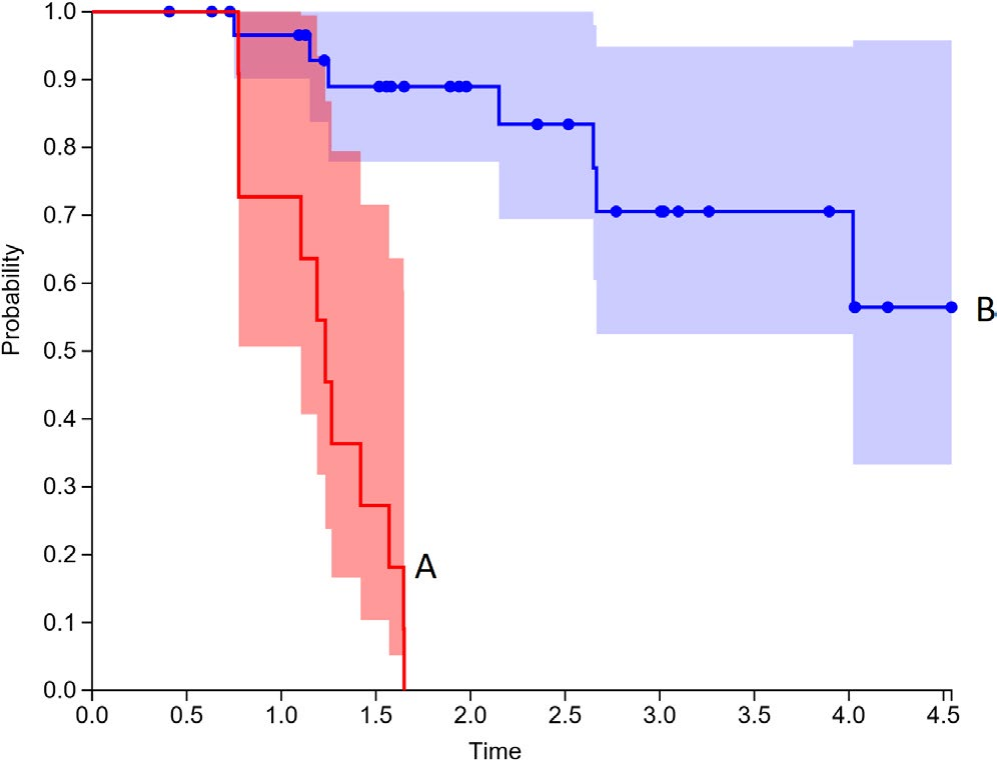
Kaplan‐Meier plot Progression Free Survival: Group A SUVmax > 5 and Group B SUVmax < 5

**FIGURE 5:**
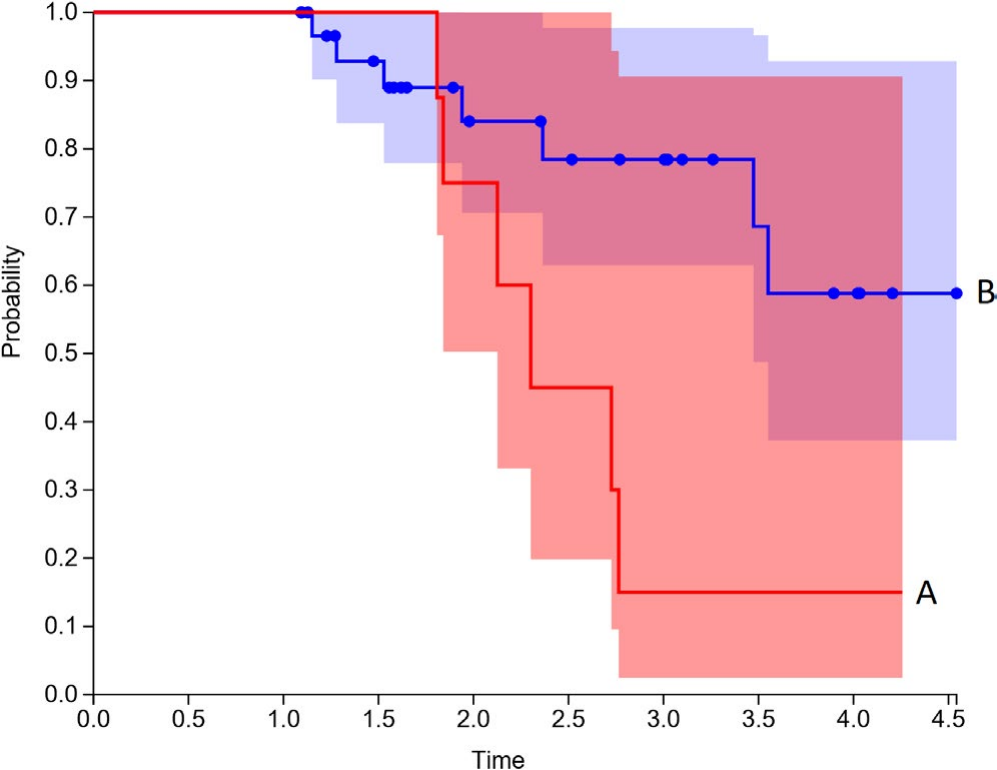
Kaplan‐Meier plot Overall Survival: Group A SUVmax > 5 and Group B SUVmax < 5

Kaplan‐Meier analysis for PET > 9 months was performed (Figures 3, 4, 5), and found that patients with SUVmax > 5 had worse local recurrence (*P* < .001), progression free survival (*P* < .001), and overall survival (*P* = .025). Median time to local recurrence, any recurrence, and death were 14 months, 14 months, and 26 months.

SUL (SUV corrected for lean body) was 27% lower than SUVmax, with a median value of 2.2 compared to 2.8. However, the difference was highly variable with a standard deviation of 13.5%. Using receiver operator curves, the optimal threshold for local recurrence was 3.8 with sensitivity of 91% (95% CI 62%‐99.8%) and specificity of 100% (89.1%‐100%).

## 4 DISCUSSION

Our study supports the body of evidence which shows a strong association for PET SUVmax and local recurrence after SBRT for NSCLC. After the acute phase (6‐9 months), SUVmax greater than 5 was highly sensitive and specific for local recurrence of NSCLC. For our study, follow‐up FDG‐PET was not routine and performed for clinical or radiological suspicion. Hence, there is a selection bias which accounts for to the high rate of reported disease progression. When taking the whole cohort into account, the 2‐year local control rate of 80% is similar to that reported by Takeda and TROG 09.02 CHISEL, but inferior to studies where patients were only treated with 3 fractions (eg, RTOG 0236, RTOG 0618).^26^, ^27^, ^28^, ^29^ The PFS and overall survival were similar to RTOG 0236, RTOG 0618, and CHISEL.^27^, ^28^, ^29^ The selection bias was anticipated and not expected to affect sensitivity or specificity. Only half of recurrences were able to be biopsy proven because of patient comorbidity, and there were no negative biopsies. For unproven recurrences, we utilized the retrospective nature of the study to have stricter longitudinal radiological criteria than RECIST, requiring progressive lesion growth over at least 3 CT scans. Thus, our unproven recurrences were unlikely to be pneumonitis, and most patients subsequently received further cancer treatments.

The timing of PET scans is important, as metabolic activity can be acutely increased either from an acute radiation pneumonitis or residual metabolic activity from dying cancer cells.^10^ A study which looked at serial FDG‐PET imaging in 19 patients after SBRT found temporary fluctuations up to a SUVmax of 5 in the first year.^18^ This activity resolves over time, and commonly SUVmax remains 2‐3 without recurrence. Our study had several patients with high SUV which resolved over time. This signal confounds early PET scans, and previous studies with PET scans at 3 and 6 months post‐SBRT have either not found an association or have lower sensitivity or specificity.^14^, ^19^, ^20^ Pierson et al studied 95 patients with 8 local recurrences, and at a median FDG‐PET timing of 3 months, found no association with SUVmax.^14^ Bollineni studied 130 patients with 6 local recurrences, and found SUVmax > 5 to be statistically significant with 15% local recurrences vs 2.2%.^19^ Pastis et al studied 88 patients with 16 local recurrences using SUV > 3, finding 50% sensitivity and 94% specificity.^20^

Delaying PET imaging after SBRT allows it to better differentiate local recurrence from postradiation change. In addition to less inflammatory signal, a later scan potentially allows for a recurrence to reach the size required for good PET sensitivity (5 cm^3^ for a static target, and larger accounting for respiratory motion blurring). For FDG‐PET scans beyond 6 months, Zhang et al (128 patients with 8 local recurrences) found that SUVmax > 5 had 100% sensitivity and 91% specificity for local recurrence.^21^ We had only 9 PET scans performed between 6 and 9 months, and found 2 false positives, at 7‐8 months, thus also similarly decreasing specificity. The theoretical competing risk of delaying PET is possible disease progression. We had 3 patients who progressed from local recurrence to mediastinal or metastatic disease, but this progression took 3‐5 months and occurred well beyond 9 months post‐SBRT (>12 months). Thus, delaying a PET scan from 6 to 9 months would increase specificity, with a theoretical risk of progression.

A range of SUVmax thresholds have been reported for local recurrence post‐SBRT PET. A systematic review and meta‐analysis conducted by Huang et al in 2012 found that recurrent disease should be suspected if high‐risk CT changes are seen with SUVmax > 5 on PET.^10^ Since then, further studies support the use of posttreatment PET SUVmax, with patient cohorts from 29 to 257 and SUVmax thresholds from 3 to 6.^22^, ^23^, ^24^, ^25^, ^26^

Obesity and lean body weight correction may explain some of the variation in SUV thresholds, but not all studies published details of their PET imaging protocol which makes it difficult to compare. Adipose tissue uptakes less FDG dose, so obesity causes higher systemic tracer levels, resulting in the lesion of interest to have a higher SUV value. We found that correction for obesity decreased SUV values by 27% on average. Our SUL threshold of 3.8 (derived by receiver operator curve minimum error) corresponds to a 25% reduction of SUV. The variation in correction was large, with the highest being a patient with a BMI of 56 who had a reduction of 73% (SUV of 1.5 vs SUL 0.4). However, diagnostic accuracy was unchanged as there was no crossover of patients over the lower threshold. We hypothesized that SUL would have better diagnostic accuracy than SUV, but this was not demonstrated in this study. Newer CT‐based lean body mass estimation techniques are more accurate than those based on height and weight formulas, and could be a further avenue of research.^30^

The retrospective nature of this study presents limitations such as incomplete information and follow‐up bias. However, we believe our findings to be consistent because all patients had PET scans in our department. There may have been some minor differences in the timing of recurrences due to nonuniformity of follow‐up between different oncologists. Interpretation bias was minimized by utilizing strict criteria for recurrences without biopsy. Our study had a shorter average follow‐up at 23.8 months compared to other studies, but remains sufficient as most local recurrences occur within the first 2 years.^8^

Future studies will be especially important for operable patients who undergo SBRT due to the possibility of salvage therapy. FDG‐PET protocols should be reported, including techniques to correct for adiposity, to more readily enable comparison of SUVmax thresholds. Defining local recurrence after SBRT is difficult on CT alone due to radiation‐induced lung opacity. Biopsy remains the gold standard, but FDG‐PET will continue to play an important role for detecting local, regional, and metastatic recurrences.

## 5 CONCLUSION

SUVmax > 5 on FDG‐PET has excellent diagnostic value when performed at least 6 to 9 months after SBRT. If suspicious CT changes are found, we recommend PET to help distinguish radiation opacities from local recurrence, and to proceed to biopsy where clinically appropriate.

## CONFLICT OF INTEREST

No potential conflict of interest was reported by the authors.

## AUTHOR CONTRIBUTIONS

Dr Daren Tan—principle author, wrote the protocol, designed the data collection tools, wrote the data analysis plan, cleaned and analyzed the data, and drafted and revised the study; Dr Suki Gill—revised the protocol, and revised the study; Dr Nelson Loh—monitored data collection and revised the study.

## Data Availability

The data that support the findings of this study are available from the corresponding author, DT, upon reasonable request.

## References

[1] McGuire S . World Cancer Report 2014. Geneva, Switzerland: World Health Organization, International Agency for Research on Cancer, WHO Press, 2015. Adv Nutr. 2016;7(2):418‐419. 26980827 10.3945/an.116.012211 PMC4785485

[2] Edge SB , Compton CC . The American Joint Committee on Cancer: the 7th Edition of the AJCC Cancer Staging Manual and the Future of TNM. Ann Surg Oncol. 2010;17(6):1471‐1474. 20180029 10.1245/s10434-010-0985-4

[3] Videtic GMM , Donington J , Giuliani M , et al. Stereotactic body radiation therapy for early‐stage non‐small cell lung cancer: Executive Summary of an ASTRO Evidence‐Based Guideline. Pract Radiat Oncol. 2017;7(5):295‐301. 28596092 10.1016/j.prro.2017.04.014

[4] Guckenberger M , Andratschke N , Dieckmann K , et al. ESTRO ACROP consensus guideline on implementation and practice of stereotactic body radiotherapy for peripherally located early stage non‐small cell lung cancer. Radiother Oncol. 2017;124(1):11‐17. 28687397 10.1016/j.radonc.2017.05.012

[5] Zhang B , Zhu F , Ma X , et al. Matched‐pair comparisons of stereotactic body radiotherapy (SBRT) versus surgery for the treatment of early stage non‐small cell lung cancer: a systematic review and meta‐analysis. Radiother Oncol. 2014;112(2):250‐255. 25236716 10.1016/j.radonc.2014.08.031

[6] Yu X‐J , Dai W‐R , Xu Y . Survival outcome after stereotactic body radiation therapy and surgery for early stage non‐small cell lung cancer: a meta‐analysis. J Invest Surg. 2018;31(5):440‐447. 10.1080/08941939.2017.1341573 28829659

[7] Senthi S , Lagerwaard FJ , Haasbeek CJ , Slotman BJ , Senan S . Patterns of disease recurrence after stereotactic ablative radiotherapy for early stage non‐small‐cell lung cancer: a retrospective analysis. Lancet Oncol. 2012;13(8):802‐809. 22727222 10.1016/S1470-2045(12)70242-5

[8] Sun B , Brooks ED , Komaki RU , et al. 7‐year follow‐up after stereotactic ablative radiotherapy for patients with stage I non‐small cell lung cancer: Results of a phase 2 clinical trial. Cancer. 2017;123(16):3031‐3039. 28346656 10.1002/cncr.30693 PMC5544582

[9] Takeda A , Kunieda E , Fujii H , et al. Evaluation for local failure by 18F‐FDG PET/CT in comparison with CT findings after stereotactic body radiotherapy (SBRT) for localized non‐small‐cell lung cancer. Lung Cancer. 2013;79(3):248‐253. 23246123 10.1016/j.lungcan.2012.11.008

[10] Huang K , Dahele M , Senan S , et al. Radiographic changes after lung stereotactic ablative radiotherapy (SABR) – Can we distinguish recurrence from fibrosis? A systematic review of the literature. Radiother Oncol. 2012;102(3):335‐342. 22305958 10.1016/j.radonc.2011.12.018

[11] Guckenberger M , Heilman K , Wulf J , Mueller G , Beckmann G , Flentje M . Pulmonary injury and tumor response after stereotactic body radiotherapy (SBRT): results of a serial follow‐up CT study. Radiother Oncol. 2007;85(3):435‐442. 18053602 10.1016/j.radonc.2007.10.044

[12] Mattonen SA , Ward AD , Palma DA . Pulmonary imaging after stereotactic radiotherapy—does RECIST still apply? Br J Radiol. 2016;89(1065):20160113. 27245137 10.1259/bjr.20160113 PMC5124920

[13] Nguyen TK , Senan S , Bradley JD , et al. Optimal imaging surveillance after stereotactic ablative radiation therapy for early‐stage non‐small cell lung cancer: Findings of an International Delphi Consensus Study. Pract Radiat Oncol. 2018;8(2):e71‐e78. 29291965 10.1016/j.prro.2017.10.008

[14] Pierson C , Grinchak T , Sokolovic C , et al. Response criteria in solid tumors (PERCIST/RECIST) and SUVmax in early‐stage non‐small cell lung cancer patients treated with stereotactic body radiotherapy. Radiat Oncol. 2018;13(1):34. 29486779 10.1186/s13014-018-0980-7 PMC5830069

[15] Brendle C , Kupferschläger J , Nikolaou K , la Fougère C , Gatidis S , Pfannenberg C . Is the standard uptake value (SUV) appropriate for quantification in clinical PET imaging? – Variability induced by different SUV measurements and varying reconstruction methods. Eur J Radiol. 2015;84(1):158‐162. 25467224 10.1016/j.ejrad.2014.10.018

[16] Degirmenci B , Wilson D , Laymon CM , et al. Standardized uptake value‐based evaluations of solitary pulmonary nodules using F‐18 fluorodeoxyglucose‐PET/computed tomography. Nucl Med Commun. 2008;29(7):614‐622. 18528183 10.1097/MNM.0b013e3282f9b5a0

[17] Dong M , Liu J , Sun X , Xing L . Prognositc significance of SUVmax on pretreatment 18F‐FDG PET/CT in early‐stage non‐small cell lung cancer treated with stereotactic body radiotherapy: a meta‐analysis. J Med Imaging Radiat Oncol. 2017;61(5):652‐659. 28266166 10.1111/1754-9485.12599

[18] Vahdat S , Oermann EK , Collins SP , et al. CyberKnife radiosurgery for inoperable stage IA non‐small cell lung cancer: 18F‐fluorodeoxyglucose positron emission tomography/computed tomography serial tumor response assessment. J Hematol Oncol. 2010;3(6). Available from: https://jhoonline.biomedcentral.com/articles/10.1186/1756‐8722‐3‐6 10.1186/1756-8722-3-6 PMC2830958 20132557

[19] Bollineni VR , Widder J , Pruim J , Langendijk JA , Wiegman EM . Residual ^18^F‐FDG‐PET uptake 12 weeks after stereotactic ablative radiotherapy for stage I non‐small‐cell lung cancer predicts local control. Int J Radiat Oncol Biol Phys. 2012;83(4):e551‐e555. 22417800 10.1016/j.ijrobp.2012.01.012

[20] Pastis NJ , Greer TJ , Tanner NT , et al. Assessing the usefulness of 18F‐fluorodeoxyglucose PET‐CT scan after stereotactic body radiotherapy for early‐stage non‐small cell lung cancer. Chest. 2014;146(2):406‐411. 24577678 10.1378/chest.13-2281 PMC4137590

[21] Zhang XU , Liu H , Balter P , et al. Positron emission tomography for assessing local failure after stereotactic body radiotherapy for non‐small‐cell lung cancer. Int J Radiat Oncol Biol Phys. 2012;83(5):1558‐1565. 22572078 10.1016/j.ijrobp.2011.10.035 PMC3474601

[22] Clarke K , Taremi M , Dahele M , et al. Stereotactic body radiotherapy (SBRT) for non‐small cell lung cancer (NSCLC): is FDG‐PET a predictor of outcome? Radiother Oncol. 2012;104(1):62‐66. 22682749 10.1016/j.radonc.2012.04.019

[23] Ebright MI , Russo GA , Gupta A , Subramaniam RM , Fernando HC , Kachnic LA . Positron emission tomography combined with diagnostic chest computed tomography enhances detection of regional recurrence after stereotactic body radiation therapy for early stage non–small cell lung cancer. J Thorac Cardiovasc Surg. 2013;145(3):709‐715. 23317944 10.1016/j.jtcvs.2012.12.024

[24] Essler M , Wantke J , Mayer B , et al. Positron‐emission tomography CT to identify local recurrence in stage I lung cancer patients 1 year after stereotactic body radiation therapy. Strahlenther Onkol. 2013;189(6):495‐501. 23609133 10.1007/s00066-013-0310-9

[25] Takeda A , Yokosuka N , Ohashi T , et al. The maximum standardized uptake value (SUVmax) on FDG‐PET is a strong predictor of local recurrence for localized non‐small‐cell lung cancer after stereotactic body radiotherapy (SBRT). Radiother Oncol. 2011;101(2):291‐297. 21889224 10.1016/j.radonc.2011.08.008

[26] Takeda A , Sanuki N , Fujii H , et al. Maximum standardized uptake value on FDG‐PET is a strong predictor of overall and disease‐free survival for non–small‐cell lung cancer patients after stereotactic body radiotherapy. J Thorac Oncol. 2014;9(1):65‐73. 24346094 10.1097/JTO.0000000000000031

[27] Timmerman RD , Hu C , Michalski J , et al. Long‐term results of RTOG 0236: a phase II trial of stereotactic body radiation therapy (SBRT) in the treatment of patients with medically inoperable stage i non‐small cell lung cancer. Int J Radiat Oncol Biol Phys. 2014;90(1):S30.

[28] Ball D , Mai GT , Vinod S , et al. Stereotactic ablative radiotherapy versus standard radiotherapy in stage 1 non‐small‐cell lung cancer (TROG 09.02 CHISEL): a phase 3, open‐label, randomised controlled trial. Lancet Oncol. 2019;20(4):494‐503. 30770291 10.1016/S1470-2045(18)30896-9

[29] Timmerman RD , Paulus R , Pass HI , et al. Stereotactic body radiation therapy for operable early‐stage lung cancer. JAMA Oncol. 2018;4(9):1263‐1266. 29852037 10.1001/jamaoncol.2018.1251 PMC6117102

[30] Kim WH , Kim CG , Kim D‐W . Comparison of SUVs normalized by lean body mass determined by CT with those normalized by lean body mass estimated by predictive equations in normal tissues. Nucl Med Mol Imaging. 2012;46(3):182‐188. 24900058 10.1007/s13139-012-0146-8 PMC4043039

